# Generation of elliptically polarized nitrogen ion laser fields using two-color femtosecond laser pulses

**DOI:** 10.1038/srep21504

**Published:** 2016-02-18

**Authors:** Ziting Li, Bin Zeng, Wei Chu, Hongqiang Xie, Jinping Yao, Guihua Li, Lingling Qiao, Zhanshan Wang, Ya Cheng

**Affiliations:** 1 School of Physics Science and Engineering, Tongji University, Shanghai 200092, China; 2 State Key Laboratory of High Field Laser Physics, Shanghai Institute of Optics and Fine Mechanics, Chinese Academy of Sciences, Shanghai 201800, China; 3 University of Chinese Academy of Sciences, Beijing 100049, China; 4 Collaborative Innovation Center of Extreme Optics, Shanxi University, Taiyuan, Shanxi 030006, China

## Abstract

We experimentally investigate generation of nitrogen molecular ion (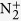) lasers with two femtosecond laser pulses at different wavelengths. The first pulse serves as the pump which ionizes the nitrogen molecules and excites the molecular ions to excited electronic states. The second pulse serves as the probe which leads to stimulated emission from the excited molecular ions. We observe that changing the angle between the polarization directions of the two pulses gives rise to elliptically polarized 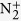 laser fields, which is interpreted as a result of strong birefringence of the gain medium near the wavelengths of the 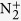 laser.

Recently, lasing action produced in population-inverted assembles, such as nitrogen/oxygen atoms^1^,^2^, nitrogen molecular ions^3^,^4^ and neutral nitrogen molecules^5^,^6^, has attracted a great deal of research interests because of its promising potentials in remote sensing applications^7^,^8^. Among them, the mechanism behind the establishment of the population inversion in neutral nitrogen molecules has been well identified, which is due to the impact excitation of neutral nitrogen molecules from the ground state to the 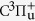 state by energetic free electrons produced in the intense laser fields^9^,^10^,^11^. However, a widely accepted model accounting for the lasing action in nitrogen molecular ions (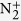) is still lacking. Yao *et al.* proposed the seed-amplification model and verified the population inversion between the 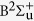 state and the 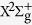 state of 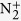 via energy amplification of a time-delayed seed pulse^3^, which was further described as a result of the couplings of the ground and two excited states of 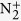 in the strong laser fields^12^. Kartashov *et al.* proposed that the different rotational periods of aligned molecular ions on the ground and excited electronic states can lead to transient laser gain and thus the creation of the coherent emissions^13^. Liu *et al.* considered the coherent emission as a super-radiant emission whose population transferred to the excited state is caused by the field-induced multiple recollisions^14^. These efforts have significantly enhanced the understanding of the physics of tunnel-ionized molecules in intense laser fields.

In this work, we report on another unusual behavior of the 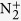 laser at the wavelength of 391 nm. Previous results show that the 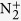 lasers induced by tunnel ionization possess the same polarization direction as that of the seed pulses when the pump and seed pulses are either parallelly or perpendicularly polarized to each other^4^. This can be well understood from a seed-amplification point of view. In such a case, the laser signal generated by the seed-amplification mechanism typically inherits all the characteristics of the seed pulses. Interestingly, when the angle between the polarization directions of the two linearly polarized pump and seed pulses is variable in the range of 0°–90°, we find that the 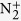 laser field becomes elliptically polarized with a variable ellipticity depending on the angle between the polarization directions of the two pulses. Moreover, the *P*-branch and *R*-branch lines in the 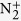 laser show dramatically different behaviors with the varying polarization direction of the seed pulses (i.e., the polarization direction of the pump is always fixed). We attempt to provide a plausible explanation to qualitatively understand this unexpected observation.

## Results

**Figure 1:**
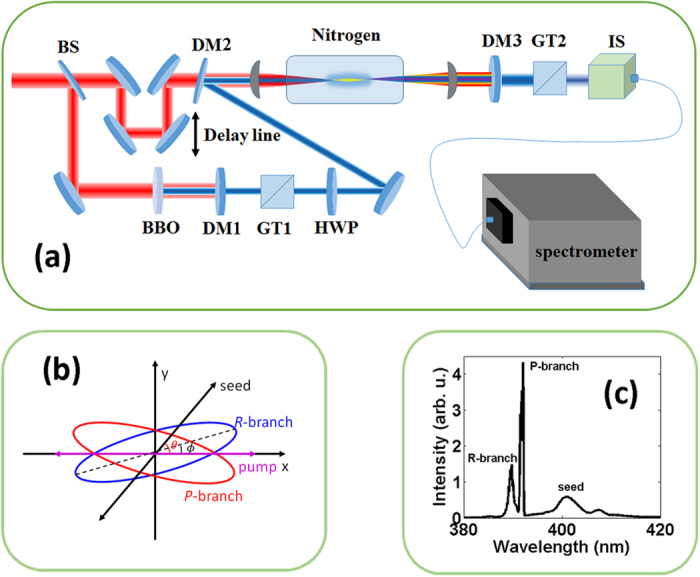
(**a**) Schematic of the experimental setup. (**b**) Polarization states of the pump pulse, the seed pulse, and the *P*-branch (red ellipse) and *R*-branch (blue ellipse) lines of the 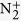 laser. (**c**) A typical spectrum of the 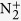 laser generated based on the seed-amplification scheme.

Our experimental setup is illustrated in Fig. 1a. The experimental details are provided in Methods. Figure 1(c) shows a typical spectrum of the 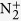 laser at 391 nm, which is assigned to the first negative band system 
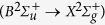
 of 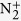. The rotational *P*-branch 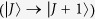 and the *R*-branch 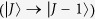 lines of the laser are labeled in the spectrum.

**Figure 2:**
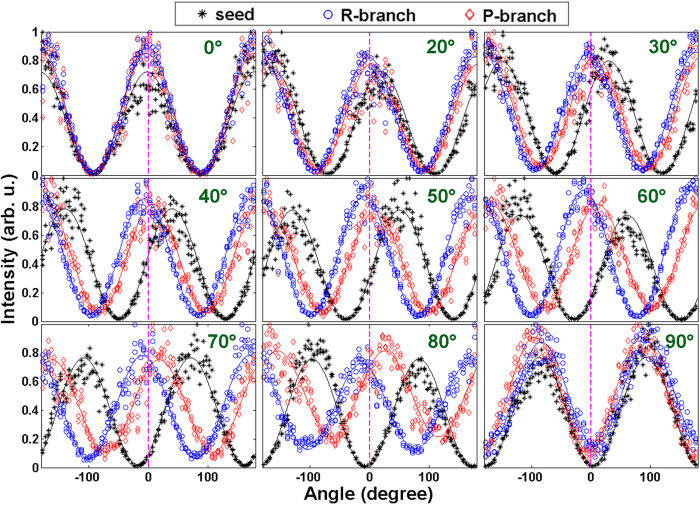
Measured intensities of the *P*-branch (red diamonds) and *R*-branch (blue circles) lines of the 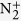 laser and the seed pulses (black stars) as functions of the angle of GT2. The angle between the polarization directions of the pump and seed pulses are indicated in each panel. The magenta dashed lines indicate the zero angle of GT2.

Figure 2 shows the intensities of the *P-*branch (red diamonds) and *R*-branch (blue circles) lines of the 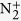 laser as well as the intensity of the seed pulses (black stars) measured after the GT2 as functions of the angle of GT2. The pressure of the nitrogen gas was 13.5 mbar and the time delay between the pump and seed pulses was set at 1.5 ps. The angle θ in the different panels of Fig. 2 are 0°, 20°, 30°, 40°, 50°, 60°, 70°, 80° and 90°. It is noticed that when the angle θ < 70°, the 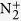 laser field is nearly linearly polarized, whereas its polarization direction is almost parallel to that of the *pump* pulses but not to that of the *seed* pulses. As the angle θ increases to 80°, the 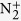 laser field becomes significantly elliptically polarized with an ellipticity of 0.28. Interestingly, the curves of *P*-branch and *R*-branch lines in Fig. 2 does not overlap, indicating their different polarization characteristics despite their wavelengths so close to each other. When the angle 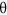 is tuned to 90°, the 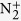 laser is linearly polarized, with its polarization direction being parallel to that of the seed pulse, which is consistent with our previous observation^4^ .

**Figure 3:**
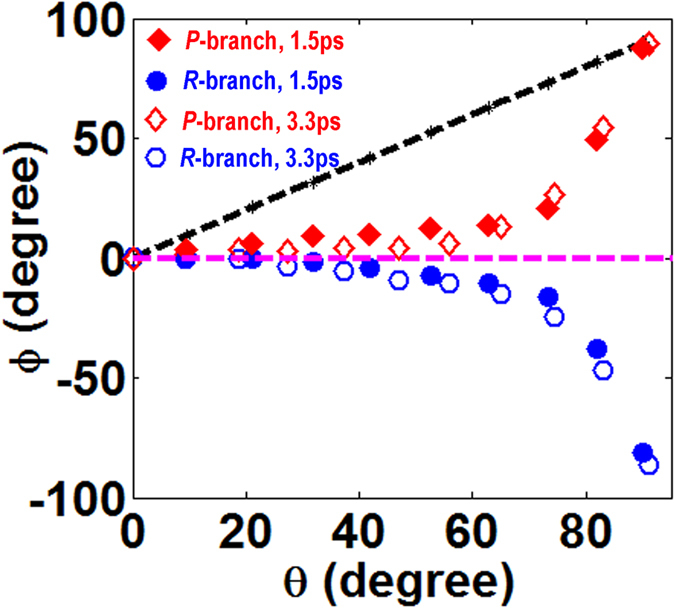
The azimuthal angle *ϕ* of the P-branch (solid diamonds) and R-branch (solid circles) lines generated at the pump-probe time delay of 1.5 ps as the functions of the angle 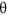. The azimuthal angle of the seed pulses (equivalent to the angle 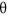 here) measured in the wavelength range of 397–410 nm (i.e., the spectral range where the laser lines are excluded) as a function of the angle 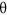 is presented with the black dashed line, showing a linear polarization unaffected by the aligned molecules. For comparison, same measurements on the azimuthal angles *ϕ* of the *P*-branch and *R*-branch lines generated at a pump-probe time delay of 3.3 ps are shown with the open diamonds and open circles, respectively.

For clarity, we plot the azimuthal angle *ϕ* of the 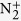 laser field generated at a time delay of ~1.5 ps as the functions of the angle of GT2 (i.e., 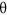) in Fig. 3. Here, the azimuthal angle *ϕ* is defined as the angle between the major axis of the 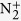 laser field and the polarization direction of the pump pulses, as indicated in Fig. 1(b). The solid diamonds represent the azimuthal angle for the *P*-branch laser lines, and the solid circles stand for the *R*-branch laser lines. It is found that the azimuthal angle of the *P*-branch lines are always positive, whereas the azimuthal angle of the *R*-branch lines are always negative. The absolute values of the azimuthal angles of both the *P*-branch and *R*-branch lines increase with the angle 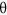.

To check how the polarization of the laser lines depends on the pump-probe delay, we changed the pump-probe time delay to 3.3 ps and performed the same measurements again. All the other parameters remain unchanged. As shown in Fig. 3 (see, the caption of Fig. 3), the data obtained at the two time delays almost overlap and only small quantitative difference is observed, indicating that the observed phenomenon is independent of the pump probe delay. We note that for both the time delays, the molecules are not at the revival times.

**Figure 4:**
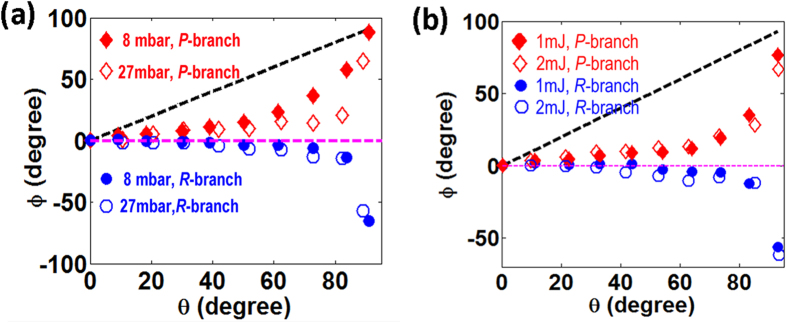
The azimuthal angles *ϕ* of the *P*-branch (diamonds), *R*-branch (circles) laser lines and the seed pulses in the wavelength range of 397–410 nm (black dashed line) as the functions of the angle 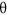 at (**a**) different gas pressures of 8 mbar (solid markers) and 27 mbar (open markers), and (**b**) different pulse energies of 1 mJ (solid markers) and 2 mJ (open markers).

Figure 4(a) compares the azimuthal angles of the *P*-branch (diamonds) and *R*-branch (circles) laser lines as the functions of the angle 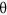 for the 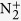 lasers generated at gas pressures of 8 mbar (solid markers) and 27 mbar (open markers) with a pump pulse energy of 2 mJ. It can be seen that at both the pressures, the azimuthal angle of the *P*-branch laser lines increases with the angle 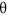, whereas the azimuthal angle of the *R*-branch decreases with the angle 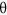. Again, the qualitative feature obtained at the different gas pressures are similar to that in Fig. 3, whereas at the higher gas pressure, the polarization states of the *P*-branch and *R*-branch lines deviate more strongly from the linear polarization of the seed.

At last, Fig. 4(b) compares the azimuthal angles of the *P*-branch (diamonds) and *R*-branch (circles) laser lines as the functions of the angle 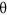 for the 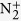 laser generated at the pump pulse energies of 1 mJ (solid marks) and 2 mJ (open marks) with a gas pressure of 13.5 mbar. The data measured at the two pump pulse energies almost overlap. It seems that the polarizations of both the *P*-branch and the *R*-branch laser lines are not sensitive to the pump pulse energy. We did not further increase the pump pulse energy, because self-seeded 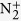 laser is generated when the pump energy is above 2 mJ. The self-seeded 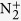 laser signal will contaminate the measurement of the dependence of polarization states of the 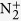 lasers on the polarization of the external seed.

## Conclusion

In conclusion, we investigated the polarization characteristics of the 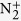 laser generated by two-color linearly polarized femtosecond laser pulses. We present an unexpected observation that the polarization of the 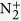 laser field does not always follow that of the seed pulses but can be controlled by changing the angle between the polarization directions of the pump and seed pulses. The *P*-branch and *R*-branch lines of the laser show different polarization characteristics as the angle between the polarization directions of the two pulses changes. Typically, in the scenario of seed amplification, the laser signal should inherit the polarization property of the seed pulses. As a result, it is commonly expected that the generated 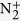 laser should always be linearly polarized and the polarization direction should be parallel to that of the seed pulses. Surprisingly, this is not the situation as evidenced by our experimental observation. Therefore, our finding indicates that propagation of ultrafast laser pulses at the resonant wavelengths of gaseous molecules has not been received sufficient attention, whose underlying physics is far from being well understood.

## Methods

### Pump-probe setup

Linearly polarized femtosecond laser beam (1 kHz, 800 nm, ~40 fs) from a commercial Ti:sapphire laser system (Legend Elite-Duo, Coherent Inc.) was divided into two with a beam splitter (BS). The first one with a pulse energy of 2 mJ was used as the pump to ionize the nitrogen molecules and build up the population inversion between the 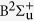 state and the 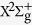 state of 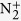. The other beam, after being frequency-doubled by a 0.2-mm thick 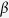-barium-borate (BBO) crystal, was used as the seed to generate the 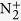 laser. The total energy of the seed pulse is ~2 μJ. As the linewidth of the N_2_^+^ laser is ~0.3 nm, the energy of the seed pulse that overlaps spatially and spectrally with the gain region is estimated to be on the level of ~100 nJ. A half wave-plate (HWP) was employed to change the polarization direction of the seed pulses. A Glan-Taylor prism (GT1) was inserted before the HWP to ensure that the seed pulses were linearly polarized. The pump and seed pulses were combined by a dichroic mirror (GM2) and focused by an *f* = 30 cm fused-silica lens into a vacuum chamber filled with nitrogen gas. The time delay between the pump and seed pulses was controlled by a motorized linear translation stage with a temporal resolution of ∼16.7 fs.

The absolute time delay between the pump and seed pulses was determined as follows. We first calibrate the absolute zero time delay by performing the sum frequency of the pump and seed pulses with a BBO. A fused silica, which has the same length of the front window of the gas chamber, was inserted before the BBO. The zero time delay was determined by maximizing the sum frequency signal. Then we reduce the length of the optical path for the pump beam by tuning the motorized linear translation stage to obtain the required time delay, i. e., 1.5 ps or 3.3 ps.

### Measurement of polarization of the 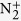 laser field

The generated 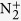 laser was first collimated by an f = 30 cm lens and then passed through a dichroic mirror (DM3) to filter out the residual pump pulses. To minimize the measurement error caused by a possible spatial anisotropy of the laser and a polarization dependent response of the spectrometer, we used an integral sphere (IS) to collect the signals and directed them into a grating spectrometer (Andor, Shamrock 303i). Another Glan-Taylor prism (GT2) was placed before the IS to measure the polarization of the laser pulses.

Throughout the experiment, we fixed the polarization direction of the pump pulses. We tuned the polarization direction of the seed pulses by rotating the HWP, and measured the intensity of the 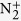 laser as a function of the angle of GT2. The zero degree of the angle of GT2 corresponds to that the optical axis of GT2 is parallel to the polarization direction of the pump pulses.

## Additional Information

**How to cite this article**: Li, Z. *et al.* Generation of elliptically polarized nitrogen ion laser fields using two-color femtosecond laser pulses. *Sci. Rep.* **6**, 21504; doi: 10.1038/srep21504 (2016).
